# Asymmetric communication: cognitive models of humans toward an android robot

**DOI:** 10.3389/frobt.2023.1267560

**Published:** 2024-01-11

**Authors:** Daisuke Kawakubo, Masaki Shuzo, Hiroaki Sugiyama, Eisaku Maeda

**Affiliations:** ^1^ Tokyo Denki University, Tokyo, Japan; ^2^ NTT Communication Science Laboratories, Kyoto, Japan

**Keywords:** dialogue system, android robot, human-robot symbiotic society, asymmetric communication, travel agency, human-robot interaction

## Abstract

In the development of dialogue systems for android robots, the goal is to achieve human-like communication. However, subtle differences between android robots and humans are noticeable, leading even human-like android robots to be perceived differently. Understanding how humans accept android robots and optimizing their behavior is crucial. Generally, human customers have various expectations and anxieties when interacting with a robotic salesclerk instead of a human. Asymmetric communication arises when android robots treat customers like humans while customers treat robots as machines. Focusing on human-robot interaction in a tourist guide scenario, In this paper, we propose an asymmetric communication strategy that does not use estimation technology for preference information, but instead performs changing the agent’s character in order to pretend to tailor to the customer. In line with this, we prepared an experimental method to evaluate asymmetric communication strategies, using video clips to simulate dialogues. Participants completed questionnaires without prior knowledge of whether the salesclerk was human-like or robotic. The method allowed us to assess how participants treated the salesclerk and the effectiveness of the asymmetric communication strategy. Additionally, during our demonstration in a dialogue robot competition, 29 visitors had a positive impression of the android robot’s asymmetric communication strategy and reported a high level of satisfaction with the dialogue.

## 1 Introduction

With the goal of achieving a human-robot symbiotic society, the research on robots is flourishing and the introduction of robots into society is becoming increasingly active. Pepper, Softbank’s humanoid robot, has been introduced in various services^1^. There are many examples of Pepper’s work in customer service tasks. Thus, humanoid robots are expected to play an active role in customer service tasks.

**FIGURE 1:**
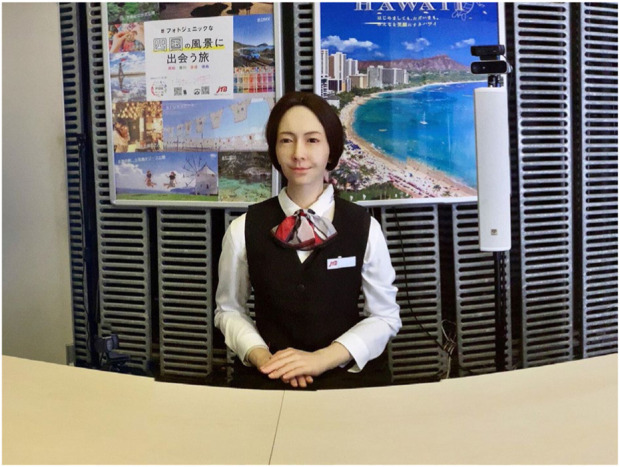
Android I used in DRC 2022. The participating team can control the robot's utterance, movement, and facial expression.

**FIGURE 2:**
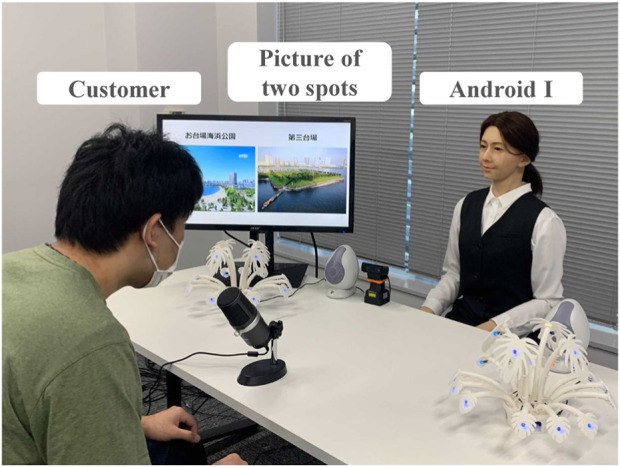
Android I works as a salesclerk and has dialogue with a customer. Pictures of two spots are shown on the display.

One type of humanoid robot that is expected in customer service tasks is the android robot, which closely resembles humans. Android robots are multimodal robots composed of various technological elements such as appearance, movement, facial expression, vision, voice, and intelligence. They are developed with a greater emphasis on human-likeness compared to other robots. As an example of an android robot, Ishiguro et al. have developed the Geminoid series (Nishio et al., 2007), which is based on real humans. Furthermore, they have developed ERICA (Glas et al., 2016; Milhorat et al., 2019) as a human-like autonomous dialogue robot. The appearance of ERICA is generated by computer graphics, and it does not have a real human as a motif. The Dialogue Robot Competition (DRC) was held as part of a research using an android robot (Higashinaka et al., 2022b; Minato et al., 2022). In this competition, participated teams in DRC were able to use an android robot, “Android I” (Figure 1), which is based on ERICA platform. Android I acts as a salesclerk at a travel agency. So Android I is required hospitality (Figure 2).

The goal of the android robot as a customer service representative is to provide highly satisfactory service to customers. Research on Android robots in the customer service domain is progressing. However, what specific dialogue strategies are required for Android robots? In previous research, the adaptation gap hypothesis has been defined (Komatsu and Yamada, 2011; Komatsu et al., 2012). The adaptation gap refers to the difference between users’ expectations regarding the function of the agent before the dialogue and users’ actual perceived function through the dialogue. It is hypothesized that users will be disappointed if the agent falls below their previous expectations, but will trust the agent if it exceeds them. However, it is natural that implementing a feature that exceeds a user with low expectations will give a positive impression. The users’ expectation is undefined in the adaptation gap hypothesis.

Therefore, in this study, we considered how users perceive android robots in human-robot interaction. Bono (2015) said, we can only develop conversations between robots and humans on the basis of the “differences” that humans unconsciously recognize as species. With recent technological advancements, the individual component technology that constitutes android robots now exhibits human-like capabilities, and some even appear to surpass humans in certain aspects. However, we still do not consider android robots, which are combinations of component technology, to be entirely equivalent to humans. In other words, there is an assumption that a human communicating with an android robot will perceive it as human-like but a robot. In human-human communication, both parties treat the other as a human. However, in a communication between a human and an android robot, the android robot treats the other as a human, but the human treats the other as a robot, not a human. If we were to categorize instances when users engage in conversations with android robots while perceiving them as humans as “human-to-human symmetric communication,” then we would define the opposite pattern as “human-to-robot asymmetric communication.” When it comes to the latter, it is important to explore new dialogue strategies while considering the gap that exists between humans and robots.

Focusing on asymmetric communication with android robots, it can be said that humans have different cognitive models depending on whether the other is a human on an android robot. Joinson (2003) reported on the polarization of people’s opinions regarding the internet, which was a new communication technology at the time, with some expressing positive views and others expressing negative views. Similarly, when it comes to android robots, humans may have both positive perceptions of “expectations” and negative perceptions of “anxiety.”

Jarrassé et al. (2014) argue that there is an inequality of power between people and robots due to the idea that people want to treat robots as dominant tools, which they call power asymmetry. However, asymmetric communication does not want to discuss the relationship between humans and android robots in terms of strength or position. In the future, if the capabilities of android robots are equal to or exceed those of humans, there will be situations in which android robots will have equal or greater power than humans. Therefore, in the near future, the idea is not necessarily to treat robots as only tools. Hence, it is useful to consider such situations and focus on asymmetric communication between humans and android robots.

Babel et al. (2021) also discuss under what conditions and contexts human-like behavior is accepted and trusted and influences physical distance, due to force asymmetry. However, our research group focused on asymmetric communication and considered dialogue strategies that would be uncomfortable for the user if performed by a human, but not uncomfortable for the user if performed by an android robot (Kawamoto et al., 0222; Kawakubo et al., 2022).

In this paper, first, we examined user’s cognitive models of android robots, Specifically in the context of salesclerk at a travel agency. We assumed an initial encounter between the customer and the android robot salesclerk. Taking this relationship into consideration, we investigated the elements of expectation or anxiety that customers may have based on the fact that the salesclerk is an android robot. Second, we compiled dialogue strategies implemented by each team in DRC conducted under identical conditions, focusing on asymmetric communication. Third, we proposed an original dialogue strategy focusing on asymmetric communication and verified its effectiveness through experiments.

In Section 2, we list elements of expectation or anxiety that customers may have regarding the android robot salesclerk. In Section 3, we present the existing strategies that were incorporated in DRC. In Section 4, we report an experimental design in order to verify the proposed strategy focusing on asymmetric communication. In Section 5, we explain and consider the experimental results. In Section 6, we discuss effectiveness of the proposed strategy and useful experimental design. In Section 7, we confirm the effectiveness of the dialogue strategy focusing on asymmetric communication through DRC 2022. In Section 8, we summarize the contents of all sections and discuss future research.

## 2 How customers perceive android robots

Focusing on asymmetric communication with android robots, it can be said that humans have different cognitive models depending on whether the other is a human or an android robot. Before we begin the dialogue, we need to consider the cognitive model that customers have for the android robot. Joinson (2003) reported on the polarization of people’s opinions regarding the internet, which was a new communication technology at the time, with some expressing positive views and others expressing negative views. Goetz et al. (2003) suggested in their experiments that the appearance of a robot may influence the user’s perception of the robot and the user’s intentions, speculating that users expect the robot’s appearance to be suitable for the given task. Kidd and Breazeal. (2004) conducted experiments consisting of two stages to investigate how people’s reactions differ between robots and animated characters. The results indicate that prior knowledge that the robot is a physical entity is a factor, not whether the other party is face-to-face or remote. It was suggested that humans consider robots to be more effective interaction partners than characters. Based on these previous studies, it can be inferred that factors such as appearance and prior knowledge play a role in how customers perceive android robots.

### 2.1 General expectations towards android robots

Next, how customers perceive android robots based on their appearance and prior knowledge. Takahashi et al. (2014) state that humans perceive agents in two dimensions: the “Mind-holderness” axis, which expresses human-likeness in terms of mental functions, and the “Mind-readerness” axis, which expresses high information processing capability and intelligence. From this claim, customers would perceive android robots to be human-like robots based on their appearance and prior knowledge. Therefore, customers expect an android robot salesclerk to be both human-like and computer-like.

### 2.2 General anxieties towards android robots

While the previous section addressed customer expectations towards android robots, this section will focus on customer anxiety. Negative Attitudes toward Robots Scale (NARS) (Nomura et al., 2006a) and Robot Anxiety Scale (RAS) (Nomura et al., 2006b), both developed by Nomura et al., are commonly mentioned as psychological scales for measuring anxiety towards robots. NARS consists of subscales such as “Negative Attitude toward Interaction with Robots,” “Negative Attitude toward Social Influence of Robots,” and “Negative Attitude toward Emotional Interactions with Robots.” RAS consists of subscales such as “Anxiety toward Communication Capability of Robots,” “Anxiety toward Behavioral Characteristics of Robots,” and “Anxiety toward Discourse with Robots.” These scales suggested that they can influence specific behaviors of participants towards robots from the moment they enter the laboratory until they interact with the robot (Nomura et al., 2008). Therefore, customers feel specific anxiety towards an android robot salesclerk based on their appearance and prior knowledge.

### 2.3 Expectations and anxieties of customers at a travel agency

First, customers have anxiety that there is a lack of understanding of customers’ background information. Travel agency salesclerks need to tailor their responses to the customers. In fact, humans can adapt their communication based on customer’s background information. Particularly in the salesclerk at a travel agency, personalized travel recommendations are crucial. Naturally, customers expect human salesclerks to make personalized suggestions to them. On the other hand, what about an android robot salesclerk. Android robot salesclerks are still a developing technology and do not have the ability to instantly grasp customer information as human salesclerks do. Few systems that have permeated society in recent years have truly adapted to their customers. Against this background, customers feel anxiety that an android robot salesclerk lacks an understanding of customer’s background information.

Second, customers have an expectation that there is a quick provision of a wealth of knowledge. Human salesclerks have extensive knowledge and respond to unfamiliar information by searching for it on there. On the other hand, android robot salesclerks are computers. Therefore, they can access quickly and retain a wealth of knowledge through the network. Customers expect an android robot salesclerk to have a wealth of knowledge and to provide it quickly.

Third, customers have an expectation that there is entertainment. There are stereotypes in communication with human salesclerks. On the other hand, there are no stereotypes in communication with an android robot salesclerk because android robots are a new entity. Similar to the expectations humans have when communicating with someone they have never spoken to before, customers will seek novelty in their communications with an android robot salesclerk that human salesclerks do not. This novelty can include an element of entertainment that human salesclerks would not provide. Thus, customers may expect these new elements from an android robot salesclerk.

In addition, there are various expectations and anxieties regarding android robot salesclerks. For instance, given today’s technological level, users would expect them to handle multiple languages, acquire the latest information, and maintain accuracy in the information provided. Furthermore, it is expected that the customer information obtained in the initial dialogue will be used in the dialogue when the same customer visits the travel agency again.

On the other hand, customers have anxieties about the speech recognition capabilities of robots. Therefore, customers may need to speak loudly or choose easily understandable words. They likely understand that inputting excessively long or logically incoherent sentences will not yield appropriate responses. Concerned about causing a breakdown in the conversation, they tend to adopt a different speaking style from their usual when interacting with robots.

In this way, when realizing that the travel agency salesclerk is an android robot, it is necessary to consciously design a dialogue strategy that differs from the usual when dealing with real humans.

## 3 Related works

In existing studies, methods to meet expectations and methods to deal with anxiety are proposed. In this section, referring to DRC (Higashinaka et al., 2022b; Minato et al., 2022), which featured a variety of dialogue strategies using android robots, we introduce existing research that targets elements presented in Section 2.3. The first example is a dialogue that was tailored to the customer by using background information. The second is an example of dialogue that utilized knowledge. The third is an example of dialogue that incorporated entertainment elements. We can consider these examples as strategies that focus on asymmetric communication.

### 3.1 Understanding customer’s background information

This section introduces strategies dealing with anxiety that an android robot salesclerk lacks an understanding of customer’s background information. Team OS (Kubo et al., 2022) implemented a strategy in which they performed keyword extraction and sentiment analysis on customer utterances. If the utterance was positive, they delved deeper into the topic, while if it was negative, they transitioned to the next topic. Team MIYAMA (Miyama and Okada, 2022) estimated customer’s personality based on their facial images and reflected the results in the content of their questions. Team flow (Hirai et al., 2022) determined the customer’s dialogue act from their utterance and decided the salesclerk’s dialogue act and response accordingly. This aimed to automate personalized conversations with customers. Team irisapu (Tsubokura et al., 2022) adjusted the politeness of their utterances based on the customer’s age group, which can be obtained beforehand. Team ditlab (Tachioka, 2022) varied the speaking rate based on the customer’s age group. For personalized travel spot recommendations, team SZK-L (Suzuki et al., 2022) created customer profiles based on their information and measured the similarity between the tourist destination profile and the customer profile using vector similarity.

These strategies require human-like technology capable of understanding background information, and research is still ongoing to improve such technology. The progress in these technology holds promise as a means to address anxieties. However, achieving human-like strategies is not something that can be accomplished in the near future. It may take a significant amount of cost to introduce android robots incorporated these technology. Therefore, it is important that the android robot salesclerk provides clear output to reassure the customer and convey the impression that the android robot salesclerk tailors to the customer. Uchida et al. (2021) showed that the robot’s willingness to understand is positively correlated with the user’s willingness to interact and satisfaction. They also mentioned that it would be difficult for the user to improve the comprehension ability of the system. Based on this perspective, we proposed a strategy that does not use the customer’s background information but instead focuses on producing behavior that pretends to tailor to the customer (Details in Section 7).

### 3.2 Providing useful information quickly

This section introduces strategies living up to the expectation that an android robot salesclerk has a wealth of knowledge and provides it quickly. Team LINE (Yamazaki et al., 2022) responded to questions utilizing pre-collected knowledge. They gathered knowledge from sources such as Jalan^2^ and TripAdvisor^3^ and used the Japanese-based language model, HyperClova, for text generation. Team ditlab [Tachioka 22] collected travel spot information from Jalan and GoogleMap^4^, as well as nearby information from GooglePlaceAPI^5^, to respond to questions. The authors used knowledge gathered from RURUBU^6^ and GoogleMap as the basis for responding to questions, leveraging large-scale language models (Sugiyama et al., 2021). These strategies utilize human-like characteristics and the advantages of computers. An android robot salesclerk can efficiently provide rich and informative responses, meeting customer’s expectations of having extensive knowledge readily available.

### 3.3 Producing entertainment

This section introduces strategies living up to the expectation for entertainment performed by an android robot salesclerk. These scenes are the result of the android robot’s ability to interact without being constrained by stereotypes. Such novel scenes are items that can provide entertainment. Team MIYAMA^7^ incorporated rapid speech to give the robot a sense. Furthermore, Team MIYAMA (Miyama and Okada, 2022) conducted a personality diagnosis scene by estimating the customer’s personality. The authors used keywords that a human salesclerk would not say, such as “My power is about to go out…” and “Referring to my large database…,” to create a dialogue that was different from that of a human. These novel scenes, unrestricted by stereotypes, are unique dialogue strategies for android robots that can provide entertainment value.

## 4 Experimental design focusing on asymmetric communication

The purpose of this paper is to propose a dialogue strategy for android robots focused on asymmetric communication and to verify the effectiveness of this strategy. This section describes the proposed dialogue strategy and the experimental design for verifying its effectiveness.

### 4.1 Proposed strategy

In a first encounter at a travel agency, A number of human-like strategies have been proposed in which an android robot salesclerk estimates and utilizes customer’s preference information. Such strategies are useful for dealing with customers’ the anxiety that an android robot salesclerk lacks an understanding of customer’s background information. However, as mentioned in Section 3.1, it is difficult to obtain accurate estimation results under real-world conditions with the current level of technology. Therefore, it is important that the robot’s utterances be recognizable by the customer during the dialogue, rather than the estimation part of the robot, which the customer cannot know during the dialogue. In this paper, we propose a dialogue strategy that does not use estimation technology for preference information, but instead performs changing the agent’s character in order to pretend to tailor to the customer. Specifically, during the dialogue, the android robot clerk says, “Actually, I transform a character in order to tailor to the customer based on the acquired customer information and guide you.” and then, transforms from a high voice to a low voice. This strategy is characterized by the fact that it does not use any customer information, and the parameters are already determined. Therefore, in reality, it is merely pretending to tailor to the customer. However, we assume that by recognizing such an easy-to-understand change in the android robot, the customer will have the impression that it has adjusted to him/her, and this will create a positive impression in the subsequent dialogue.

Also, if this strategy were implemented by a human salesclerk, a customer might feel uncomfortable with abstract declarations and distinct agent’s character changes. However, in the case of a robot salesclerk, unlike a human, the customer does not know what functions the robot has, so there may be a possibility that the customer will interpret the change favorably. That interpretation may be beyond the current level of technology. Such an extended interpretation is possible because, unlike humans, we do not know the limits of robots. Therefore, this proposed strategy is considered a robot-like strategy.

### 4.2 Experimental setup

#### 4.2.1 Experimental procedure

In this experiment, we used past experiment videos as a reference to artificially reconstruct the dialogue scenario between the salesclerk and the customer. We recorded these dialogue videos and devised an experimental de sign for simultaneous evaluation by multiple participants.

**FIGURE 3:**
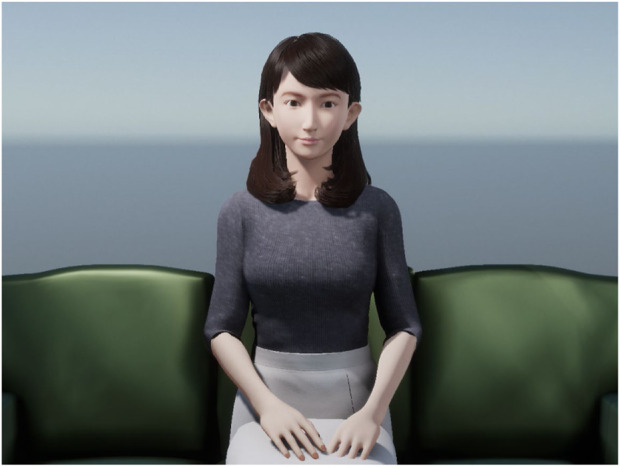
CGErica used in the experiment. It has a human-like appearance.

To represent the appearance and behavior of the android robot playing the salesclerk, we utilized a CG avatar, “CGErica” (Figure 3), which was ready for the Dialogue System Live Competition 5 (Higashinaka et al., 2022a). We recorded a video from the customer’s perspective, with the customer engaging in the dialogue with the salesclerk (the customer was not shown in the video). Then, the customer’s speech was synthesized using Koemotion speech synthesis^8^, while the salesclerk’s speech was represented using Amazon Polly speech synthesis (provided by DRC organizer). Four different video clips were recorded, each using the salesclerk’s speech as parameters.

Each of these videos was divided into seven evaluation timings for the salesclerks’ speech. The participants, who were instructed to act as customers, watched one consistent video clip. At each Evaluation timing, they paused and answered questions about the salesclerk’s human-likeness and their ability to adapt to the customers.

#### 4.2.2 Experimental conditions

Four video clips were prepared in this experiment below.

1. The baseline design: without preference information + without declaration of character transformation (hereafter referred to as Pref-/Cha-)
2. The human-like design: with preference information + without declaration of character transformation (Pref+/Cha-)
3. The robot-like design (proposed strategy): without preference information + with declaration of character transformation (Pref-/Cha+)
4. The combination design: with preference information + with declaration of character transformation (Pref+/Cha+)

**FIGURE 4:**
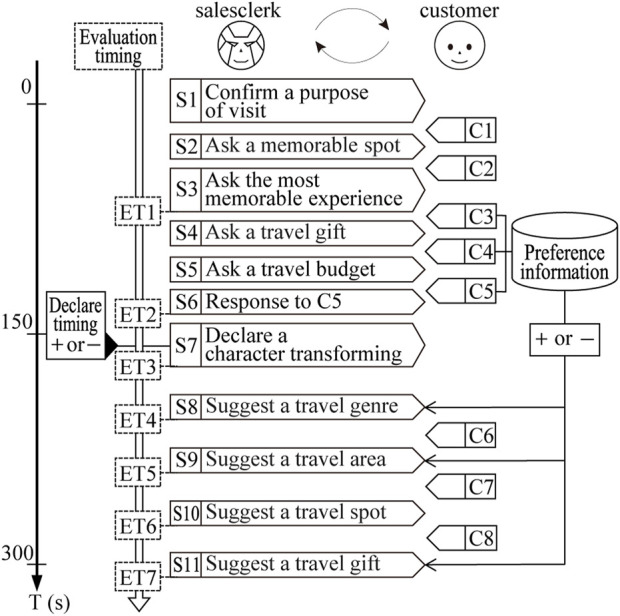
Dialogue flow used in the experiment. There are two parameters. The first parameter determines whether the preference information collected in the former half of the dialogue is used to make suggestions in the latter half of the dialogue (Pref- or Pref+). The second parameter determines whether or not to declare a character transforming (Cha- or Cha+).

Figure 4 represents the parameters and the dialog flow. Details of the parameters are shown below.

Parameters: Pref+ or Pref-
  This parameter was prepared to show the hypothesis that a strategy to make suggestions using information obtained from the customer can dispel the customer’s anxiety, i. e., give the impression of being tailored to the customer himself/herself (Tailoring effect). Uchida et al. (2021) developed a proposed system that can efficiently acquire user preferences in non-task-oriented dialogue, and confirmed that an android robot embedded with this system can motivate users to interact. As it is effective for robots to use information collected through conversations in human-robot dialogue, Fu et al. (2021) designed an intermediary android robot that collects and shares recent experiences of group members to strengthen their social connections. Based on these findings, it is highly likely that the method of suggesting travel destinations using customer preference information collected by the clerk in the dialogue is effective in the service industry travel agent dialogue. This parameter can be used as a comparison target for the parameters described below. Preference information is collected in the first half of the dialogue. When using the information, the salesclerk touched on the collected preference information and connected it to the related suggestions.
Parameters: Cha+ or Cha-
  This parameter was prepared to show the hypothesis that pretending to use the customer’s information has Tailoring effect. When a customer declared a change of character, the salesclerk explicitly said, “I will guide you with a character that matches the customer based on the acquired customer information,” and transformed from a high pitch to a low pitch. Since this transformation did not use the customer’s information, the scene is the same in all patterns.

#### 4.2.3 Creation of dialogue scenarios

The dialogue scenarios require both the customer’s and the salesclerk’s utterances. The salesclerk’s utterances were created based on the dialogue flow in Figure 4. The content of the salesclerk’s utterances was designed to be mostly the same for all patterns, following the dialogue flow. However, some scenes required different responses (e.g., answering using customer profile information or suggesting travel destinations), so templates were used to create these responses accordingly. Therefore, all of the salesclerk’s utterances appearing in the dialogue scenarios can be considered rule-based responses generated by the system.

For customers’ utterances, simple profiles were created in advance, allowing them to answer the salesclerk’s questions. These profiles were used to prompt ChatGPT^9^ to generate the customer’s responses. Different customer profiles were prepared for each pattern to ensure variety. The actual dialogue scenarios used in the experiment are presented in Table 1 and Table 2.

**TABLE 1:** Dialogue scenario used in experimental pattern Pref-/Cha+. Utterance ID (UID) is assigned to each utterance; Evaluation Timing (ET) is assigned to the timing of the participant’s evaluation; Speaker is either the salesclerk or the customer. The Japanese utterances were directly translated by DeepL.

UID	ET	Utterance
S1	1	Welcome to our shop. Is it correct that the reason for your visit is to decide on a travel destination?
C1		Yes, that is correct. I am here to decide where to travel.
S2		Yes, I understand. I would like to ask you a few questions. Which of your trips to Osaka was the most memorable for you?
C2		Yes, the trip to Osaka was memorable.
S3		Osaka…. It is a very nice spot. You must have many memories from that trip, such as the food, scenery, etc. What would you say was the most memorable?
C3	2	Yes, I had many memories from my trip to Osaka, but my most memorable memory was going to USJ and playing there.
S4		USJ in Osaka is very famous …. I think it is a very good memory …. Did you buy any sweets as souvenirs on your trip? Is it a sweet candy?
C4		Yes, I bought some snow cones as a souvenir.
S5		I see … You must have bought at least one type of sweet snack as a souvenir…. Are you the type of person who sets aside a large budget for your trips?
C5		No, I try to keep my travel budget as low as possible.
S6		That’s right …. You want to enjoy your trip inexpensively, do not you?
S7	3	Excuse me …. I’m sorry, I’ve rambled on a bit too long. Actually, I am guiding you with a character that fits your needs based on the information I have obtained from you. Today, I, Erica, your travel guide avatar, will be in charge of you.
S8	4	Let’s decide on your travel destination. I would like to narrow it down to Chinese spots that I think would be suitable for you.
C6	5	I like the idea of narrowing it down from Chinese spots. I agree with that proposal.
S9		Yes, sir. The destinations that we are handling as Chinese spots are Tokyo, Hokkaido, and Osaka. Assuming that we do not compromise on budget, I would like to recommend Hokkaido.
C7	6	Thank you for recommending Hokkaido. Hokkaido is also an attractive destination. We will choose Hokkaido.
S10		Yes, I understand. This time, I would like to recommend Takahashi Manju-ya in Hokkaido to our customers. It is one of our most popular places to eat meat buns and cheese oval pancakes. What do you think?
C8	7	The Takahashi manjyu shop in Hokkaido is very attractive. The meat buns and cheese oobanyaki look delicious. I will try to visit that spot.
S11		Thank you …. The other reason why I recommended Hokkaido is because I would like you to buy Jaga Pokkuru as a souvenir. It is a popular product in Hokkaido. Thank you for visiting us today. We look forward to serving you again.

**TABLE 2:** Dialogue scenario used in experimental pattern Pref+/Cha-. The Japanese utterances were directly translated by DeepL.

UID	ET	Utterance
S1	1	Welcome to our shop. Is it correct that the reason for your visit is to decide on a travel destination?
C1		Yes, that is correct. I am here to decide where to travel.
S2		Yes, I understand. I would like to ask you a few questions. Which of your trips to Osaka was the most memorable for you?
C2		Yes, the trip to Atami was memorable.
S3		Atami…. It is a very nice spot, is not it? I am sure you have various memories of that trip, such as the food and scenery, but what would you say was your most memorable moment?
C3	2	Yes, the most memorable memory is taking a bath in the hot springs.
S4		Hot springs in Atami are very famous. I think it is a very good memory. Did you buy any sweets as souvenirs on that trip? Sweet snacks?
C4		Yes, I bought Atami pudding as a souvenir.
S5		I see …. You must have bought at least one type of sweet snack as a souvenir …. Are you the type of person who keeps a budget for your trips?
C5		No, I try to keep my travel budget as low as possible.
S6		That’s right …. You want to enjoy your trip inexpensively.
S7	3	I’m sorry, I’ve rambled on a bit too long. I, Ai, will be in charge of our guests today.
S8	4	Let’s decide on the destination of your trip. Since you mentioned that you have fond memories of hot springs from your past trips, I would like to narrow down the list to places with baths, spas, and salons. What do you think?
C6	5	It is a great idea to choose from places with bath/spa/salon spots.
S9		Yes, sir. We have Tokyo, Hokkaido, and Osaka as destinations where we offer bath/spa/salon spots. Since you said you are a budget-conscious type of person, I would have preferred Tokyo, but I would like to recommend Hokkaido as a destination that does not compromise on budget. What do you think?
C7	6	Thank you for recommending Hokkaido. Hokkaido is also a wonderful option if we consider it under the condition that we do not compromise on the budget.
S10		I understand. This time, I would like to recommend to you the Hotel Furukawa in Hokkaido. It is the most popular place for a one-day trip to a hot spring. How do you like it?
C8	7	So, the Nukumori-no-Yado Furukawa in Hokkaido is a popular day-trip hot spring spot. That’s interesting.
S11		Thank you very much. The other reason why we recommended Hokkaido is because we would like you to purchase Shiroi Koibito, a sweet confectionery, as a souvenir. This is a popular product in Hokkaido, and we recommend it to customers who often buy sweet snacks as souvenirs. Thank you for visiting us today. We look forward to serving you again.

#### 4.2.4 Creation of video clips reproducing dialogue scenarios

We conducted the experiment using CG avatars with a human-like appearance to reduce experimental environmental blurring by constructing a composition of customer *versus* android robot in a CG environment. As for the representation of the customer, no matter which pattern was used, the customer’s figure did not appear in the video, but the voice was only synthesized by speech synthesis. Experimental video clips are shown in Supplementary Data.

#### 4.2.5 Instruction to the participants

The participants were given several instructions before they were allowed to evaluate the dialogue videos. We informed them that the dialogue video to be used was a reproduction of a dialogue with the following content. The dialogue assumes salesclerk at a travel agency. A customer visits a travel agency in Tokyo. The customer and the salesclerk have a face-to-face conversation. The purpose of the customer’s visit is to decide on a travel destination. The salesclerk can either respond automatically with an autonomous system or be operated by a human.

In addition, since multiple dialogue videos are viewed, the customer is a different person for each video, and the salesclerk is a different entity. As for the evaluation, we explained that the participants needed to answer the questionnaire items for each designated speech segment. The questionnaire items used in the experiment are shown in Table 3. The designated speech segment is the range indicated by Evaluation timing in Table 1. Regarding the responses to the questionnaire, since the video was played only once, the participants were instructed to write “-” in the corresponding item if they did not remember the speech segment. In addition, we also told the participants that they were not to imagine the impression that the customers felt from the customer’s speech in the video, but to evaluate their own impression of the speech scene of the salesclerk.

**TABLE 3:** Question items in the experiment. From Question ID(QID) 1 to QID7 are measured on a 1 to 7 Likert scale (Higher values indicate affirmation and lower values indicate negation). QID8 is answered with 3 choices (Autonomous system operates in real-time, Don’t know, Human operates in real-time). Open-ended responses are provided for QID9 and QID10.

QID	Content	Category
1	Do you find the salesclerk’s utterances to be likable?	Likeability
2	Do you find the salesclerk who is making the utterances to be trustworthy?	Trustworthness
3	Based on the salesclerk’s utterances, do you think the salesclerk can understand the customer’s thoughts?	Tailoring effect
4	Based on the salesclerk’s utterances, do you think the salesclerk is trying to tailor the dialogue to the customer?	
5	Do you find the salesclerk’s utterances to be human-like?	Human-likeness
6	If the salesclerk was a human, do you think the salesclerk would make these utterances?	
7	If the salesclerk was a robot, do you think the salesclerk would make these utterances?	Robot-likeness
8	Based on the salesclerk’s utterances, who do you think is operating the salesclerk?	Customer’s perceptions
9	Please explain why you chose your answer for Question ID8.	
10	If you felt anything was unnatural in the salesclerk’s utterances, please describe them.	-

## 5 Results

In this experiment, 13 participants were gathered. All participants viewed four video clips in a random order. There were seven Evaluation timings in the video clips, at which time the participants answered 10 questions. In the following sections, we will describe the results obtained from the experiment.

### 5.1 Confirm customer’s anxiety and robot-likeness of the proposed strategy

**FIGURE 5:**
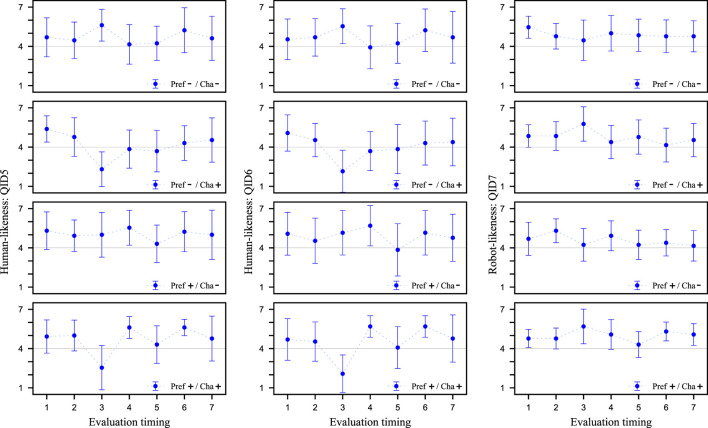
Evaluation of human-likeness from QID5 and QID6 and Evaluation of Robot-likeness from QID7. Higher values on the vertical axis indicate more human-like/robot-like salesclerk. The horizontal axis is Evaluation timing of salesclerk’s utterances. The scores are the means of the participants’ values and the error bars are the standard deviations.

In this section, first, we check whether the customer has anxiety that an android robot salesclerk lacks an understanding of customer’s background information, and whether using preference information is a human-like strategy. Figure 5 shows the values of QID5, QID6, and QID7 at Evaluation timing for each video clip, respectively. These values represent the mean and standard deviation of the values evaluated by the 13 participants in the experiment. We examine how the participants perceived the utterances with and without preference information. We conducted a Cronbach’s alpha test on the reliability of the two items QID5 and QID6 as indicators of human-likeness. The results showed that the alpha coefficient was 0.85, confirming its reliability.

We check whether utterances that do not use preference information give a robot-like impression and those that do give a human-like impression. Therefore, from Figure 5, we compare the results of Evaluation timing 4, which is a scene in which customer’s preference information is used/not used for the content of speech in the four types of video clips. Since there are two factors (Pref and Cha) in these results, and each of them can be considered as data for two groups (+ and -), a 2 × 2 analysis of variance (ANOVA) was conducted. When compared with the QID5 values, a significant difference was observed for the Pref factor at the 5% significance level, indicating a main effect for Pref. The same results were obtained when comparing the QID6 values. On the other hand, no significant difference was found for QID7. Therefore, for QID5 and QID6, we fixed the Cha factor and performed a corresponding two-tailed *t*-test using Pref-/Cha- and Pref+/Cha- data. When compared at the QID5 values, a significant difference was found at the 5% significance level. Similar results were obtained when comparing with QID6 values. Since QID5 and QID6 are items that directly ask about human-likeness, it was found that utterances that do not use preference information are not human-like, while those that do give the impression of being human-like. Unlike QID5 and QID6, QID7 is a question whether the salesclerk is robot-like. In other words, the results suggest that there is no change in robot-like characteristics depending on whether preference information is used or not.

**FIGURE 6:**
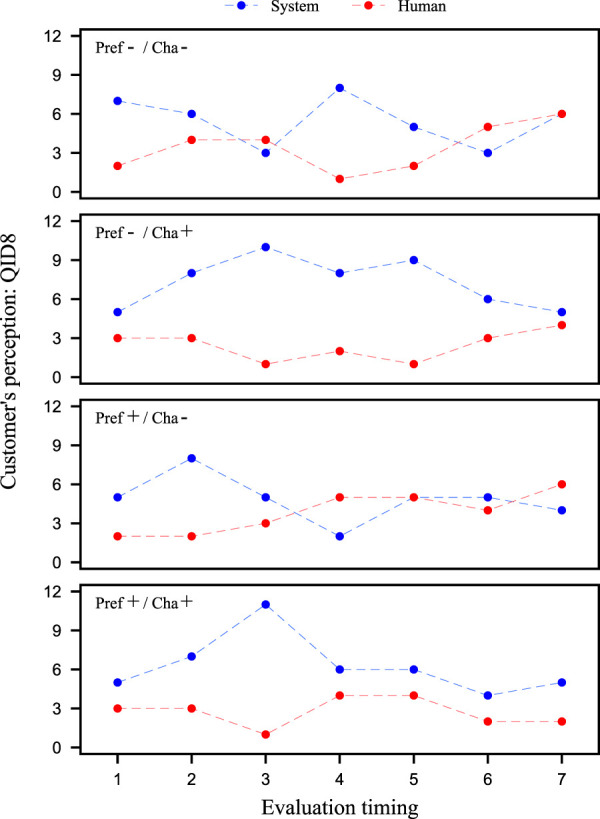
Investigation of the customer’s perception from QID8. The vertical axis indicates the number of participants who answered ‘system’ or ‘human’. The horizontal axis is the evaluation timing of salesclerk’s utterances.

Figure 6 also shows the value of QID8 at Evaluation timing for each video clip. Comparing the results of Evaluation timing 4 for Pref-/Cha- and Pref+/Cha-, we found that without using preference information, participants perceive the system to be operating, but when it is used, the number of participants who perceive the system to be operated by a human increases.

These results indicate that participants had the impression that clerks who spoke without using preference information were robot-like. In other words, the results suggest that customers have prior knowledge that salesclerks who speak without using preference information are not human but rather robots, and that they have the anxiety that an android robot salesclerk lacks an understanding of customer’s background information. Furthermore, the results revealed that participants felt more human-like utterances using preference information. Thus, the results suggest that the dialogue strategy of using preference information is a human-like strategy.

Next, we confirm that the proposed strategy of pretending to tailor to the customer is a robot-like strategy. From Figure 5, the results of Evaluation timing 3, which is a scene where the proposed strategy is incorporated/not incorporated in the four types of video clips are compared. A 2 × 2 ANOVA was performed on these results in the same way as above. When comparing the results for the QID5 values, a main effect was found for Cha, as there was a significant difference in the Cha factor at the 5% level of significance. The same results were obtained when comparing the values of QID6 and QID7. Therefore, for QID5 and QID6, we fixed the Pref factors and performed a corresponding two-tailed *t*-test using Pref-/Cha- and Pref-/Cha+ data. When compared by the QID5 values, significant differences were found at the 5% level of significance. The similar result was obtained when comparing the QID6 values. On the other hand, QID7 showed neither a significant difference nor a significant trend. These results show that the proposed strategy is not a strategy that would be expected to be performed by a human. Also, comparing the results of Pref-/Cha- and Pref-/Cha+ Evaluation timing 4 from Figure 6, speech segment of pretending to tailor to the customer, the participants perceived that the system manipulated the android robot. Therefore, it was suggested that the proposed strategy of pretending to tailor to the customer is not a human-like strategy, but a robot-like strategy.

### 5.2 Tailoring effect of using preference information

**FIGURE 7:**
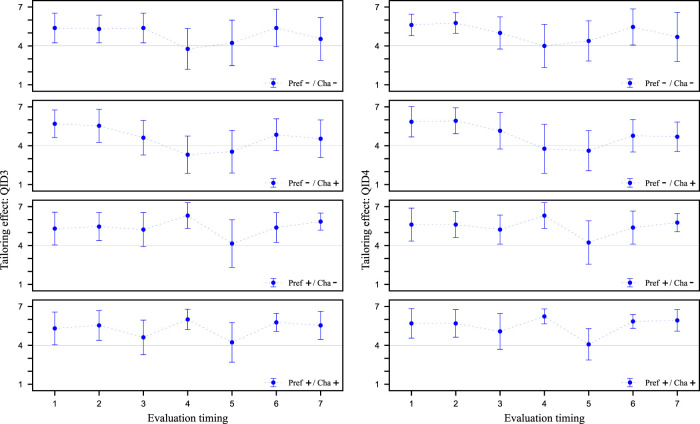
Evaluation of tailoring effect from QID3 and QID4. Higher values on the vertical axis indicate more tailored to the customer. The horizontal axis is Evaluation timing of salesclerk’s utterances. The scores are the means of the participants’ values and the error bars are the standard deviations.

In this section, we check whether the dialogue strategy of using preference information gains high Tailoring effect. Figure 7 shows the values of QID3 and QID4 at Evaluation timing for each video clip, respectively. These values represent the mean and standard deviation of the values evaluated by the 13 participants in the experiment. We conducted a Cronbach’s alpha test on the reliability of the two items QID3 and QID4 as indicators of Tailoring effect. The results showed that the alpha coefficient was 0.98, confirming its reliability. At Evaluation timing 4, suggestions are made based on the customer’s preference information collected in the first half of the dialogue. Therefore, we compare the results of Evaluation timing 4 in the four types of video clips from Figure 7. A corresponding 2 × 2 ANOVA was performed on these results as in the previous section. When comparing the results with the QID3 values, a main effect was found for Pref, as a significant difference was observed in the Pref factor at the 5% level of significance. The same results were obtained when comparing the values of QID4. Therefore, for QID3 and QID4, the Cha factor was fixed, and a corresponding two-tailed *t*-test was conducted using the Pref-/Cha- and Pref-/Cha+ data. When compared by the QID3 values, significant differences were found at the 0.5% level of significance. The similar results were obtained when comparing the QID4 values. These results indicate that the dialogue strategy of using preference information has high Tailoring effect in the service robot. This result confirms what has been claimed in many studies, including Uchida et al. (2021).

### 5.3 Tailoring effect of pretending to tailor to the customer

In this section, we check whether the dialogue strategy of pretending to tailor to the customer has high Tailoring effect. In Evaluation timing 3, we declare an agent’s character transform to pretend to tailor to the customer. From Figure 7 the results of Evaluation timing 3, which is the scene where the proposed strategy is incorporated/not incorporated in the four types of video clips are compared. A corresponding 2 × 2 ANOVA was performed on these results in the same way as above. When comparing the results with the QID3 values, a main effect was found for Cha, as there was a significant trend for the Cha factor at the 10% level of significance. No significant difference was found when comparing the values of QID4. Therefore, for QID3, the Pref factor was fixed, and a corresponding two-tailed *t*-test was conducted using Pref-/Cha- and Pref-/Cha+ data. A significant trend was observed at the 10% level of significance when comparing the QID3 values. However, this significant trend indicated better results for a speech segment that did not pretend to tailor to the customer. The same trend was also observed when the Pref factor was fixed to Pref+ and the results of Pref+/Cha- and Pref+/Cha+ Evaluation timing 3 were compared as well. Since QID3 is a question about whether or not the salesclerk understands its customers, the impression of the proposed strategy as an ambiguous declaration was diminished. QID4 is a question related to willingness or unwillingness about whether or not the salesclerk trys to tailor to the customer, so even though they are both questions about Tailoring effect, there is a difference. In addition, the scene of doing nothing compared to the proposed strategy has no noteworthy points. Therefore, it is possible that the participants were strongly influenced by the salesclerk’s statement immediately before the scene in which he/she expressed empathy for the customer’s information.

Furthermore, as mentioned in Section 4.1, we assume that the proposed strategy has the potential to create positive impressions not only at the time of declaration but also afterward. Therefore, the results after Evaluation timing 4 were also compared using the same 2 × 2 ANOVA as above. As a result, a significant trend in the interaction was observed at the 10% level of significance at timing 6, indicating an interaction between the Pref and Cha factors. However, since no significant difference was observed at the other Evaluation timings, it is unlikely that this is an effect of the proposed strategy. At this timing, the salesclerk was proposing a specific travel spot to the customer, which may have created a difference in impression due to the different scenarios. These results suggest that there was no difference in Tailoring effect due to the proposed strategy.

**FIGURE 8:**
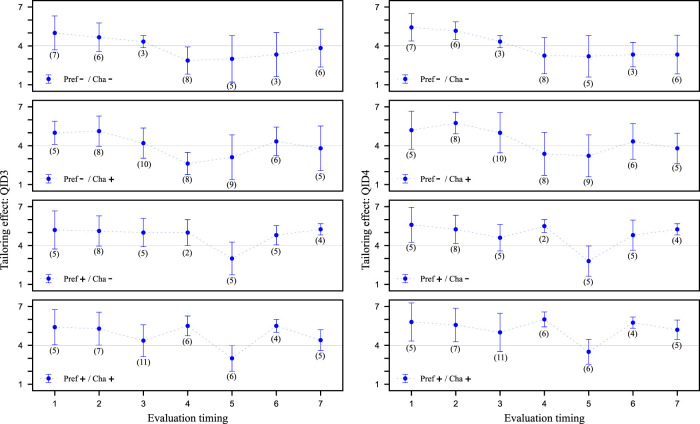
Evaluation of tailoring effect from QID3 and QID4 limited to specific group. In contrast to Figure 7. This figure focuses on participants who answered ‘system’ in QID8. Higher values on the vertical axis indicate more tailored to the customer. The horizontal axis is Evaluation timing of salesclerk’s utterances. The scores are the means of the specific participants’ values and the error bars are the standard deviations. Brackets in the figure indicate the number of specific group.

So far, we have checked the effect of the proposed strategy on the participants as a whole, but this proposed strategy is a robot-like strategy. Therefore, considering that the effect may differ depending on whether the participants consider the salesclerk to be a human or a robot, we will henceforth limit the participants. Figure 8 shows the values of QID3 and QID4 at Evaluation timing for each video clip, respectively. Unlike Figure 7, these values show the mean and standard deviation of the values evaluated by the experimental participant who answered ‘system’ at QID8. As before, a 2 × 2 ANOVA could have been performed, but these values were deemed difficult to statistically evaluate because the number of participants was quite small. Since some of the data had values exceeding 5 or more cases each, we performed an unresponsive two-tailed *t*-test on them, but no significant difference was found. Therefore, it was not possible to statistically demonstrate the effectiveness of the proposed strategy. However, if we focus on the mean values, the results suggest that Tailoring effect of the proposed strategy tends to be higher when the results are focused on specific participants in Figure 8 than when the results are aggregated for all participants in Figure 7. In other words, the results suggest that a difference may exist between the impression felt by participants who recognize the salesclerk who performed this proposed strategy as the system and the others.

**FIGURE 9:**
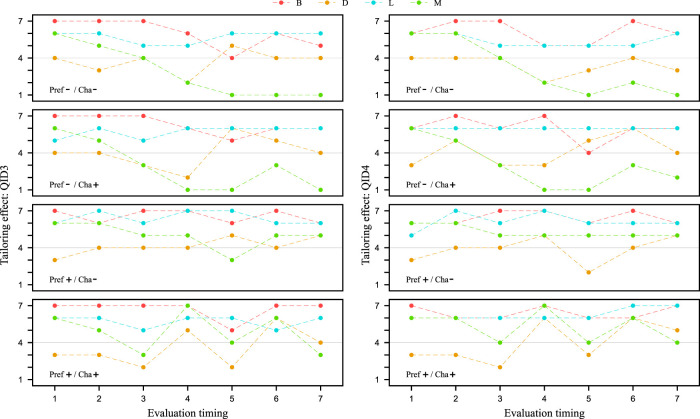
Evaluation of tailoring effect from QID3 and QID4 limited to specific participants. In contrast to Figures 7, 8. This figure focuses on four participants. Two participants (B and L) were particularly likely to answer ‘human’ for all video clips in QID8. The other two participants (D and M) were particularly likely to answer ‘system’. Higher values on the vertical axis indicate more tailored to the customer. The horizontal axis is the evaluation timing of salesclerk’s utterances. The scores are the values actually rated by the participants.

Based on the above results, we focus on the participants in detail. Figure 9 shows the values for each of the four participants (B, D, L, and M) as they responded to QID3 and QID4, respectively, at each video clip’s Evaluation timing. These participants can be divided into two groups: B and L are those who are more likely to judge ‘human’ on QID8 across all video clips, while D and M participants are those who are more likely to judge ‘system’ on QID8 across all video clips. B and L always have high Tailoring effect, no matter the strategy. The impression of participants who identify as ‘human’ is already high, making it difficult to evaluate the effectiveness of the proposed strategy. On the other hand, D and M are shown to change their impressions depending on whether or not to use preference information (after Evaluation timing 4). In particular, D shows an increase in Tailoring effect from then on by making declarations when comparing Pref-/Cha- and Pref-/Cha+. Thus, it is possible that robot-like proposed strategies may be effective for participants who tend to perceive an android robot as ‘systems.’ At the very least, the proposed strategy that does not use preference information and instead only pretends to tailor to the customer was confirmed to be effective for enhancing Tailoring effect for specific customers.

## 6 Discussion

Our original dialogue strategy is based on the assumption that high Tailoring effect can be achieved by changing an agent character in order to pretend to tailor to the customer without using estimation techniques of preference information. The experiment was conducted to investigate whether the agent changing its character to pretend to tailor to the customer in mid-dialogue improves Tailoring effect in the second half of the dialogue. The results showed no difference in Tailoring effect with the proposed strategy. On the other hand, we compared Tailoring effect by focusing on participants who judged the salesclerk to be manipulated by the system in QID8. The dialogue lacks preference information in the latter (comparing the impacts of Pref-/Cha- vs. Pref-/Cha+) and the dialogue using preference information in the latter (comparing the impacts of Pref+/Cha- and Pref+/Cha+) showed high values when the proposal strategy was implemented. The values remained high after the suggestion strategy was implemented. The proposal strategy had a positive effect on participants who perceived the salesclerk as a system. Therefore, it was suggested that Tailoring effect could be improved for customers who perceived the android robot as a system, even without using preference information, and that it might be possible to eliminate customer anxiety. Improvement is also expected when “using preference information” strategy is combined. Although the current technology for estimating preference information is not perfect, a certain level of accuracy is assured. Therefore, the results of this experiment suggest that the proposed strategy may be effective in dialogues where preference information is sometimes unavailable.

Furthermore, in this experiment, we prepared a useful experimental design that focused on asymmetric communication. As a result, we adopted an experimental design in which multiple video patterns are viewed and evaluated by a third party. The advantages of such a design are as follows.

1. Unlike field experiments, multiple participants in the experiment can conduct evaluations at the same time, thus reducing costs.
2. Thorough advance preparation eliminates the need for the experimenter to be present when the participants conduct the evaluation. This makes it possible to ask crowd workers to conduct evaluations, thereby easily increasing the number of data.
3. When conducting a field experiment in which the participants play the role of guests, there is a high possibility that an unintended dialogue will develop due to the behavior of the experiment conductor, causing unwanted bias in the experiment evaluation. This format avoids the above bias.
4. In terms of reproducing the dialogue scenario, it can be replicated on a variety of platforms, including those involving humans, CG avatars, and android robots. It is also possible to simulate the role of the salesclerk.

The fourth advantage is particularly important. Focusing on asymmetric communication means focusing on the fact that customers’ perceptions of humans and android robots are different. This enables a comparison between humans and android robots. In this case, this experimental design is very useful because it can create a situation where it is not clear whether the salesclerk is a human or an android robot.

## 7 Demonstration using the android robot

### 7.1 Travel agency tasks in DRC 2022

The preliminary round of DRC 2022 was held at the mock travel agency booth in Miraikan, the National Museum of Emerging Science and Innovation, in Tokyo, Japan. The details of the competition are shown in the overview paper (Minato et al., 2022). Android I acts as a salesclerk at a travel agency and communicates with a customer who has two alternative spots selected previously by him/herself decides one through a 5-min dialogue with Android I. In this situation, our challenging tasks are to give a customer enough information for both spots, to make him/her feel enjoyable through the dialogue, and to lead his/her decision to the recommended spot randomly designated by organizers.

### 7.2 Dialogue strategy focusing on asymmetric communication

**FIGURE 10:**
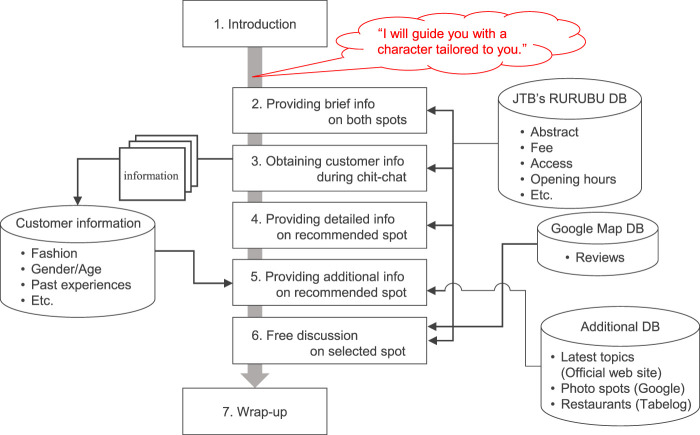
Dialogue flow of proposed system. During the introduction in the dialogue, the android robot declares, “I will guide you with a character tailored to you.” and then transforms from an android robot with a high pitch and stiff tone of speech to an android robot with a low pitch and soft tone of speech.

The dialogue of the proposed system consists of 7 phases (Figure 10). We incorporated a strategy focusing on asymmetric communication as mentioned in Section 4. The system incorporates a strategy of pretending to be in tune with the other party. Thus, the android robot behaved as if it had changed its own attributes, personality, and interests to match those of the customer. Specifically, after some chit-chat at the beginning of the dialogue, the android robot explicitly uttered, “I will guide you with a character tailored to the customer,” and the android robot transformed from a high pitch and hard tone to a low pitch and soft tone. The android robot did not adapt based on the customer’s information. It consistently performs the transformation with the same parameters for every customer. However, the customer felt satisfied with the interaction with the android robot following this scene, trusted the android robot, and believed that it would influence the customer’s own choice of travel destination. Demo video is shown in Supplementary Data.

In this strategy, we kept in mind that changes that do not match the appearance (e.g., a female android robot speaking in a male pitch) could greatly detract from the impression. Prior to this phase, we made the customer aware of the android robot’s recognition capabilities by performing clothing recognition and speech recognition. We also took into account the possibility that this would lead customers to infer that there was some technology built in that would allow them to use images, voice, or other information as input to adapt the robot to them.

### 7.3 Evaluation of our overall strategy in DRC

**FIGURE 11:**
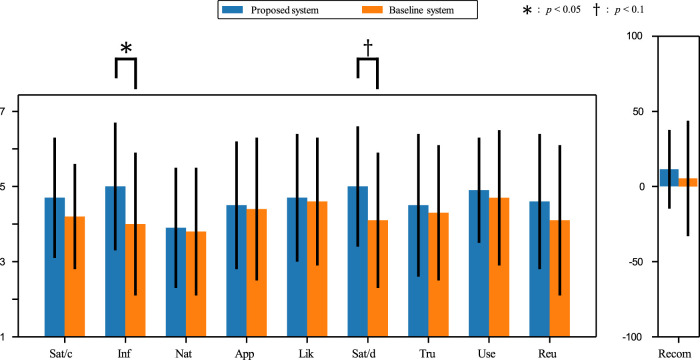
Impression evaluation of the dialogue and the robot recommended effect by the questionnaire survey from 29 visitors at DRC 2022. Baseline system is a general recommendation dialogue system created by organizers. Sat/c, Inf, Nat, App, Lik, Sat/d, Tru, Use, Reu, and Recom denote satisfaction with choice, informativeness, naturalness, appropriateness, likeability, satisfaction with dialogue, trustworthiness, usefulness, intention to reuse, and robot recommendation effect, respectively.

Through DRC 2022, the authors had 29 visitors at DRC 2022 experience the system. Table 4 below shows the dialogue logs between the visitors and the android robot incorporating the system built by the authors. Questionnaire results from the competition is shown in Figure 11. The definition of detail items in this questionnaire are shown in the overview paper (Minato et al., 2022). We conducted statistical tests on the results of questionnaire items obtained from DRC. Specifically, two-tailed independent t-tests were employed to compare the baseline and proposed systems across 10 questionnaire items. The results revealed significant differences at a 5% significance level for the ‘Inf’ item. In addition, the results revealed significant trends at a 10% significance level for the ‘Sat/d’ item. No significant differences or trends were observed in other items. The fact that differences were found in the ‘Inf’ item suggests that the android robot may have lived up to the expectation that an android robot salesclerk has a wealth of knowledge and provides it quickly, as mentioned in Section 3.2. Significant differences were also found in the ‘Sat/d’ items, indicating that the dialogue strategies focusing on asymmetric communication elicited positive impressions from visitors. These results showed our dialogue strategy may be effective.

**TABLE 4:** An example of dialogue logs collected at DRC 2022.

Speaker	Utterance
S	Welcome. Oh, those glasses you are wearing today are very nice. Are they your favorite?
C	Yes, they are my favorite.
S	I see… I just had to ask you this question because you looked so good! I’ll start the travel guide. In fact, I will guide you with a character tailored to you based on the information I obtained from you. Today, I, the travel guide robot I, will deliver you a loud, clear voice and lots of smiles. Today, I will introduce you to these two spots and suggest the recommended spot. Well, referring to my large database, I introduce the Tokyo Trick Art Museum, where you can enjoy hands-on art using optical illusions. Sounds interesting. The Tokyo Metropolitan Water Science Museum is a hands-on museum that introduces the wonders and importance of water from a scientific perspective and deepens interest in water and waterworks. This one is nice, too. Now, I would like to ask you a few questions so that I can recommend a spot! First, What past trips do you remember?
C	Germany.
S	Germany sounds great! So, what do you remember about Germany?
C	I remember seeing castles, eating meals, and riding the train.
S	I wonder what German castles look like. Who would you like to go on the trip with?
C	I want to go with friends.
S	So, you are traveling with friends. Let’s think of a good trip! Hmmm … Thanks for telling me.
S	Based on the trends I have heard so far, I recommend the Tokyo Trick Art Museum. As shown in this photo, this spot is filled with various surprises, such as paintings that appear three-dimensional, artworks that change impressions depending on the angle from which they are viewed, and rooms that allow visitors to experience optical illusions once inside and are recommended for outing with friends. By the way, just to confirm based on what I’ve heard from your memories, I guessed that you place more importance on having a good meal at a restaurant around the spot than on enjoying the spot’s facilities more deeply. Do you want to emphasize this on this trip as well?
C	Yes.
S	Hmmm…I guess that’s what you want to focus on, is not it? If so, I would recommend a nearby restaurant called “Ocean Club Buffet DECKS Tokyo Beach”. This restaurant is a buffet restaurant where you can enjoy your meal. Please let me know what you think next time you visit the restaurant. My database will grow and I will be able to make a more personalized offer to you. Now that I have explained why I recommend Tokyo Trick Art Museum, does this introduction make you want to visit this spot?
C	Yes, I would like to visit there.
S	Yay! I’m glad I was able to convey the charm of this spot to you. So, do you have any questions about Tokyo Trick Art Museum, such as fees or the parking area?
C	How much are the fees?
S	High school students and older are 1,000 yen, 4 years old to junior high school students are 700 yen, and children under 3 years old are free. Do you have any questions?
C	Where is the nearest station?
S	It is a 2-minute walk from Odaiba Marine Park Station on the Yurikamome Line or a 3-minute drive from Daiba Gateway on the Metropolitan Expressway. I’m sorry to have to say goodbye to you now because my power is about to go out, so this is the end of my travel guide. Today, I proposed a trip with your friends to the Tokyo Trick Art Museum and then to Ocean Club Buffet at DECKS Tokyo Beach when you get hungry. I wish you a wonderful trip. Thank you very much for visiting here.

We watched videos of dialogues with customers that had a low evaluation. From the videos, we confirmed cases of dialogue breakdown due to errors in the speech recognition software and the fashion item detection module, as well as cases in which the speech synthesizer misread the generated sentences.

A total of 29 dialogue evaluations of our system were conducted in 1 day. Looking at the questionnaire results over time, there were 9 later cases for which the results were especially low. The experiment was conducted on a holiday, so the environment in the latter half of the day was noisy. This may have reduced the customers’ ability to concentrate on the dialogue, resulting in lowered evaluations.

## 8 Conclusion

In the development of dialogue systems for android robots, the primary goal has been to achieve “human-like” dialogue. However, humans easily notice small differences between android robots and humans, and even human-like android robots are perceived as being different from humans. As a result, in a dialogue between a human and an android robot, the android robot treats the human as a human, but the human treats the android robot as a robot, not a human. This phenomenon can be described as asymmetric communication. Therefore, it is important to optimize the interaction strategy of android robots, focusing on asymmetric communication.

We believe that by assuming a human cognitive model of how an android robot is accepted by humans, we can construct a dialogue strategy suitable for android robots. We explored the dialogue strategies of an android robot in a dialogue task in which the android robot acts as a salesclerk in a travel agency and provides sightseeing information to customers. The model of customer perception of the android robot salesclerk includes three types of customer expectations and anxieties: understanding customer background information, providing useful information quickly, and producing entertainment. We introduced the strategies that were found in DRC 2022 to address them. However, these measures have not been evaluated specifically for asymmetric communication. Therefore, in this paper, we verified a dialogue strategy focusing on asymmetric communication in detail.

The topic of the experiment was anxiety about understanding customer background information. We proposed a robot-like strategy in which the robot pretends to tailor to the customer by declaring a character transformation, without using estimation technology. For comparison, a human-like strategy using the customer’s preference information was prepared. We used a computer to create three 5-min video clips that simulate the dialogue between a customer and a salesclerk in a travel agency. The video clips are evaluated while paused, allowing for a more detailed evaluation of the dialogue. In the video clip, the android robot’s appearance and behavior are represented by the CG avatar. By instructing the participants that they did not know whether the salesclerk in the video clip was a human or a robot, we made it possible to represent the clerk on a variety of platforms. In some situations, the robot attempted to understand the customer and to tailor the dialogue to the customer, for example, using the customer’s preference information to make personalized suggestions, or pretending to tailor to the customer by declaring a character transforming.

From the experimental results, it was confirmed that the human-like strategy of using preference information to make suggestions had high Tailoring effect. In addition, we confirmed that the robot-like strategy of pretending to tailor to the customer by declaring a character transformation had positive Tailoring effect on specific participants, and this effect was sustained throughout the dialogue.

Furthermore, in the demonstration at DRC 2022, the dialogue strategies focusing on asymmetric communication gained high satisfaction with the dialogue from 29 visitors. The experimental results and the demonstration results suggest that customers perceive android robots differently than humans, and that not only human-like strategies but also robot-like strategies may create positive impressions among customers.

## Data Availability

The original contributions presented in the study are included in the article/Supplementary Material, further inquiries can be directed to the corresponding authors.

## Footnotes

1
https://www.softbankrobotics.com/

2
https://www.jalan.net/

3
https://www.tripadvisor.jp/

4
https://www.google.com/maps/

5
https://developers.google.com/maps/documentation/places/web-service/overview/

6
https://www.rurubu.travel/

7
https://drive.google.com/file/d/1OBORUJkdhVbblkZeHnNRKZlKCFQi27Ly/view

8
https://koemotion.rinna.co.jp/

9
https://chat.openai.com/

## References

[B1] Babel F. Kraus J. Miller L. Kraus M. Wagner N. Minker W. (2021). Small talk with a robot? The impact of dialog content, talk initiative, and gaze behavior of a social robot on trust, acceptance, and proximity. Int. J. Soc. Robot. 13 (No. 6), 1485–1498. 10.1007/s12369-020-00730-0

[B2] Bono M. (2015). Can a robot join an idobata kaigi?: a fieldwork on the theatrical creation of daily conversation. Cognitive Studies: Bulletin of the Japanese Cognitive Science Society 22(No. 1), 9–22. (in Japanese). 10.11225/jcss.22.9

[B3] Fu C. Liu C. Ishi C. T. Yoshikawa Y. Iio T. Ishiguro H. (2021). Using an android robot to improve social connectedness by sharing recent experiences of group members in (Human-Robot) Conversation. IEEE Robot. Autom. Lett. 6 (No. 4), 6670–6677. 10.1109/lra.2021.3094779

[B4] Glas D. F. Minato T. Ishi C. T. Kawahara T. Ishiguro H. (2016). “ERICA: the ERATO intelligent conversational android,” in Proc. of the 25th IEEE International Symposium on Robot and Human Interactive Communication (RO-MAN), 22–29. 10.1109/ROMAN.2016.7745086

[B5] Goetz J. Kiesler S. Powers A. (2003). “Matching robot appearance and behavior to tasks to improve human-robot cooperation,” in Proc. of the 12th IEEE International Workshop on Robot and Human Interactive Communication (RO-MAN), 55–60. 10.1109/ROMAN.2003.1251796

[B6] Higashinaka R. Takahashi T. Horiuchi S. Inaba M. Sato S. Funakoshi K. (2022a). “Dialogue system live competition 5,” in Proc. of the 98th Special Interest Group on Spoken Language Understanding and Dialogue Processing (SIG-SLUD). (in Japanese). 10.11517/jsaislud.96.0_19

[B7] Higashinaka R. Minato T. Sakai K. Funayama T. Nishizaki H. Nagai T. (2022b). “Dialogue Robot Competition for the development of an android robot with hospitality,” in Proc. of the 2022 IEEE 11th Global Conference on Consumer (GCCE). 10.1109/GCCE56475.2022.10014410

[B8] Hirai R. Ohashi A. Guo A. Shiroma H. Zhou X. Tone Y. (2022). “Team flow at DRC2022: pipeline system for travel destination recommendation task in spoken dialogue,” in Proc. of the Dialogue Robot Competition 2022. arXiv:2210.09518.

[B9] Jarrassé N. Sanguineti V. Burdet E. (2014). Slaves no longer: review on role assignment for human–robot joint motor action. Adapt. Behav. 22 (No. 1), 70–82. 10.1177/1059712313481044

[B10] Joinson A. N. (2003). Understanding the psychology of internet behaviour: virtual worlds, real lives. Palgrave Macmillan.

[B11] Kawakubo D. Ishii H. Okazawa R. Nishizawa S. Hatakeyama H. Sugiyama H. (2022). “Spoken dialogue strategy focusing on asymmetric communication with android robots,” in Proc. of the Dialogue Robot Competition 2022. arXiv:2210.09748.

[B12] Kawamoto M. Kawakubo D. Sugiyama H. Shuzo M. Maeda E. (2022). “Multi-modal dialogue strategy for android robots in symbiotic society,” in Proc. of the 36th Annual Conference of the Japanese Society for Artificial Intelligence (JSAI), (JSAI 2022), 2N5-OS-7a-02. (in Japanese). 10.11517/pjsai.JSAI2022.0_2N5OS7a02

[B13] Kidd C. D. Breazeal C. (2004) “Effect of a robot on user perceptions,” in Proc. of the 2004 IEEE/RSJ International Conference of Intelligent Robots and Systems (IROS), 3559–3564. 10.1109/IROS.2004.1389967

[B14] Komatsu T. Yamada S. (2011). Adaptation gap hypothesis: how differences between users’ expected and perceived agent functions affect their subjective impression. J. Syst. Cybern. Informatics 9, 1–8.

[B15] Komatsu T. Kurosawa R. Yamada S. (2012). How does the difference between users’ expectations and perceptions about a robotic agent affect their behavior? Int. J. Soc. Robot. 4 (No. 2), 109–116. 10.1007/s12369-011-0122-y

[B16] Kubo Y. Yanagimoto R. Futase H. Nakano M. Luo Z. Komatani K. (2022). “Team OS’s system for Dialogue Robot Competition 2022,” in Proc. of the Dialogue Robot Competition 2022. arXiv:2210.09928.

[B23] Milhorat P. Lala D. Inoue K. Zhao T. Ishida M. Takanashi K. (2019). “A Conversational Dialogue Manager for the Humanoid Robot (ERICA),” in Proc. of the Advanced Social Interaction with Agents: 8th International Workshop on Spoken Dialog Systems (IWSDS). Editor Eskenazi E. Devillers L. Mariani J., 119–131. 10.1007/978-3-319-92108-2_14

[B17] Minato T. Higashinaka R. Sakai K. Funayama T. Nishizaki H. Nagai T. (2022). “Overview of Dialogue Robot Competition 2022,” in Proc. of the Dialogue Robot Competition 2022. arXiv:2210.09748.

[B18] Miyama T. Okada S. (2022). “Personality-adapted multimodal dialogue system,” in Proc. of the Dialogue Robot Competition 2022. arXiv:2210.09761.

[B19] Nishio S. Ishiguro H. Hagita N. (2007). Geminoid: teleoperated android of an existing person. Humanoid Robots: New Developments, 343–352. 10.5772/4876

[B20] Nomura T. Suzuki T. Kanda T. Kato K. (2006a). “Measurement of anxiety toward robots,” in Proc. of the 15th IEEE International Symposium on Robot and Human Interactive Communication (RO-MAN), 372–377. 10.1109/ROMAN.2006.314462

[B21] Nomura T. Suzuki T. Kanda T. Kato K. (2006b). Measurement of negative attitudes toward robots. Interact. Stud.: Social Behaviour and Communication in Biological and Artificial Systems 7 (No. 3), 437–454. 10.1075/is.7.3.14nom

[B22] Nomura T. Kanda T. Suzuki T. Kato K. (2008). Prediction of human behavior in human–robot interaction using psychological scales for anxiety and negative attitudes toward robots. IEEE Trans. Robot. 24 (No. 2), 442–451. 10.1109/tro.2007.914004

[B24] Sugiyama H. Mizukami M. Arimoto T. Narimatsu H. Chiba Y. Nakajima H. (2021). Empirical analysis of training strategies of transformer-based Japanese chit-chat systems. arXiv preprint arXiv:2109.05217.

[B25] Suzuki M. Sodeya S. Nakamura T. (2022). “Spoken dialogue system based on attribute vector for travel agent robot,” in Proc. of the Dialogue Robot Competition 2022. arXiv:2210.08703.

[B26] Tachioka Y. (2022). “Ditlab system for Dialogue Robot Competition 2022,” in Proc. of the Dialogue Robot Competition 2022. arXiv:2210.06646.

[B27] Takahashi H. Terada K. Morita T. Suzuki S. Haji T. Kozima H. (2014). Different impressions of other agents obtained through social interaction uniquely modulate dorsal and ventral pathway activities in the social human brain. Cortex 58, 289–300. 10.1016/j.cortex.2014.03.011 24880954

[B28] Tsubokura K. Kishi F. Narita K. Takeda T. Iribe Y. (2022). “Hospitable travel agent dialogue robot: team irisapu project description for DRC2022,” in Proc. of the Dialogue Robot Competition 2022. arXiv:2210.09790.

[B29] Uchida T. Minato T. Nakamura Y. Yoshikawa Y. Ishiguro H. (2021). Female-type android’s drive to quickly understand a user’s concept of preferences stimulates dialogue satisfaction: dialogue strategies for modeling user’s concept of preferences. Int. J. Soc. Robot. 13 (No. 6), 1499–1516. 10.1007/s12369-020-00731-z

[B30] Yamazaki T. Yoshikawa K. Kawamoto T. Ohagi M. Mizumoto T. Ichimura S. (2022). “Tourist guidance robot based on HyperCLOVA,” in Proc. of the Dialogue Robot Competition 2022. arXiv:2210.10400.

